# Domestication and exposure to human social stimuli are not sufficient to trigger attachment to humans: a companion pig-dog comparative study

**DOI:** 10.1038/s41598-024-63529-3

**Published:** 2024-07-08

**Authors:** Anna Gábor, Paula Pérez Fraga, Márta Gácsi, Linda Gerencsér, Attila Andics

**Affiliations:** 1 Department of Ethology, Eötvös Loránd University, Budapest, Hungary; 2 Neuroethology of Communication Lab, Department of Ethology, Eötvös Loránd University, Budapest, Hungary; 3 ELKH-ELTE Comparative Ethology Research Group, Budapest, Hungary; 4 ELTE NAP Canine Brain Research Group, Budapest, Hungary

**Keywords:** Developmental biology, Evolution

## Abstract

Dogs exhibit human-analogue attachment to their owners, with similar function and mechanisms to that of infant-mother bond, but its origin is unclear. Comparative studies on socialised wolves and dogs emphasise genetic influence in dogs' preparedness for attachment to humans. We aimed to reveal if this genetic effect stems from general domestication or artificial selection that increased dogs' dependence on humans. We assessed and compared behavioural patterns of young companion pigs and dogs using a Strange Situation Test. Dogs but not pigs exhibited distinct behaviours towards their owner and a stranger along attachment-specific variables, so only dogs’ relevant behaviours fulfilled attachment criteria. From the observed behaviours, three factors were formed: Attachment (to the owner), Anxiety (in a strange situation), and Acceptance (of a stranger). Results indicate (1) higher Attachment scores in dogs than pigs, (2) greater Acceptance scores in pigs, (3) positive correlation of Attachment and Anxiety in both, (4) similar time tendency of pigs' Attachment and Acceptance scores. These suggest that in pigs, domestication and early exposure to human social stimuli did not trigger attachment to humans. Thus, along with species predispositions, the unique dog-owner attachment can be facilitated by artificial selection that increased dogs' dependence on humans.

## Introduction

Exploring the social relationships between humans and their companion animals is key to understanding the development and nature of interspecific socio-cognitive skills. Dogs (*Canis familiaris*) exhibit human-analogue attachment towards their owner^1^ with similar function^2^ and certain mechanisms^3^ to that of infant-mother relationships. There is growing research interest on how such a particular relationship can evolve. Even though the human social niche—in which human-animal relationships can be realised—is clearly needed for the development of attachment bonds towards the owner, previous studies on differently socialised adult^4^ and puppy^5^ dogs showed negligible environmental effects on the dog-owner bond. In line with this, differences in the human-oriented bond between dogs and wolves raised in a similar human social environment support the role of genetic changes (that domestic dogs have undergone since the dog-wolf lineage split) in dogs' preparedness to form attachment with humans (e.g.^2,5^). Although there is some debate about the extent to which the relationship between intensely socialised wolves and dogs with their owners is similar^6,7^, it is important to remark that no studies demonstrated attachment in wolves that meet with all of its operational criteria^8^. To find out if the genetic effects supporting dog–human attachment are caused by domestication in general or more specifically by the artificial selection that increased dogs' dependence on humans^9^, one should also investigate whether similarly kept individuals of other domesticated species are attached to their owners. Beyond dogs, however, cats are the only companions whose social bond to the owner has been investigated to date, but cat results are contradictory (see^10,11^, but see^12,13^) and the only direct comparison of dogs and cats using the same standard procedure did not provide evidence for cats’ attachment towards their owner^13^.

Recently, miniature variants of domestic pigs (*Sus scrofa domesticus*) (later referred to as pigs) turned out to be popular companion animals despite originally being selected for other purposes (i.e. for meat stock^14^, and later for medical research^15^). Similarly to dogs, pigs are group-living animals^16^ that, unlike cats, have a similar role in human families to that of dogs when kept as companions. While their species predispositions differ, these similarities still make companion pigs a more feasible comparison to dogs than other species living in human families. But whether companion pigs exhibit attachment behaviours towards their owner is unknown.

While 'attachment' is a broad term frequently used as a synonym for various special bonds in general psychology and in animal behaviour literature, here, under ‘attachment’, we refer to a specifically defined phenomenon that enables us to draw strict parallels between companion animal-owner and infant–mother attachment bonds. According to this definition, ‘attachment’ is an organisational construct belonging to a behavioural system^17^ that regulates long-lasting asymmetrical social relationships^18^ in which the attached individual depends on a security-providing attachment figure^19^. The function of attachment is to enhance the attached individual’s chance for survival and learning through keeping it in the proximity of the attachment figure, therefore it plays an important role in infant-mother^20^ as well as in dog-owner relationships (e.g.,^2,21,22^). Attachment is associated with reward-related neural mechanisms^3,23^, can develop early in life and is relatively stable in time in case of both humans (e.g.,^24,25^) and dogs^26^. Attachment can be assessed along objectively measurable behavioural patterns^20,27^. Specifically, these behaviours are (1) contact- and proximity-seeking with the attachment figure during exploration (secure base effect) and in case of danger (safe haven effect), (2) separation distress-related behaviours in the absence of the attachment figure, and (3) specific behavioural changes upon reunion. Importantly, all these above operational criteria must be present in an attachment relationship. While various special bonds are reported between individuals of different species (e.g.,^28^) and even towards inanimate objects (e.g., human children^29^, adults^30^, non-human primates^31^)—some characterised by behaviours also crucial in the attachment system—, the dog-owner relationship stands out as the only non-intraspecific bond that is proven to simultaneously fulfil all criteria of attachment in accordance with the definition described above^2,4,32–34^. Companion dog-owner attachment is also unique^8^ in terms of the fact that dogs can form attachment towards their owners both in their natural environment (within the human social niche) and during adulthood^2^. Studies investigating the social bond in non-human primates towards humans typically test hand-raised or captive animals, and mostly focus on non-adult subjects (e.g.,^35^).

The Strange Situation Test (SST)—a validated laboratory test (e.g.,^36^) with various precursors in the examination of social bonds across species^37^—is widely used to investigate attachment towards human individuals both intraspecifically (i.e. infant-mother relationships, e.g.,^38^) and interspecifically (e.g., dog-owner relationships^39–41^). The SST has not yet been used, however, to test pigs.

Domestic pigs, compared to dogs, exhibit various different, but also many similar human-oriented behaviours. For instance, research on farm pigs revealed pigs’ willingness to make social contact with humans (e.g.,^42–45^) already from a young age (e.g.,^44^), as has also been reported in dogs^9^. Furthermore, recent comparative studies on young companion pigs and dogs kept in a similar human social niche revealed that the two species orient towards and seek contact with humans to a similar degree in various situations^46,47^. But, as compared to dogs, the available knowledge on pigs’ interspecific social competence and affinity is still scarce.

In this comparative study, using the SST context, we investigated whether young companion pigs show attachment towards their owner, and whether pigs’ and dogs’ attachment-specific behaviours differ towards the owner and the stranger to a similar extent. We hypothesised that (H1) if domestication in general, along with socialisation in the human environment contributes to the emergence of attachment towards a human owner in social species, then intensely socialised companion pigs and dogs will behave differently with the owner and the stranger to a similar degree. Conversely, (H2) if the specific selection that has led to increased dependence on humans in dogs is a main genetic factor for interspecific attachment to arise, then only dogs and not pigs will show the specific behaviour patterns that fulfil the criteria of attachment. To investigate the temporal stability of pigs’ behaviour in the SST, we tested pigs twice, at the age of ~ 9 and ~ 18 months.

## Methods

### Participants

In this comparative study, we tested companion pigs (*Sus scrofa domesticus*) and dogs (*Canis familiaris*) living in human families in a comparably similar social environment. Pigs, as well as dogs, were considered family members and were exposed to similarly close human contact from the age of ~ 8 weeks.

Twelve juvenile companion pigs participated in the study (5 neutered females, and 7 neutered males, Minnesota and mixed miniature variants). To make pigs’ rearing environment highly similar to that of well-socialised family dogs, pigs’ adoption process was supervised and guidelines were provided by the Neuroethology of Communication Lab (ELTE, Budapest, Hungary). During this, owners were taught how to handle pigs at home, socialise them to humans, expose and habituate them to different environments and transportation, etc. To investigate the temporal stability of pigs’ behaviour, pigs were tested twice, at the age of 8.75 ± 3.64 months and 17.69 ± 2.57 months. At the time of the first test, pigs lived together with their owners for approximately 5–6 months. One pig entered the research program a few months older (at the age of 14 months) than the others. Due to a possible order effect, this pig’s test was considered as a first, and it was tested only once. One pig was used to pilot the protocol, thus its first (pilot) SST was left out of the analyses. The second test of a third pig is also missing as before the second test this subject died. As a result of these, 11 pigs participated in the first SST and 10 pigs participated in the second SST. We had a total of 12 pig subjects, as individuals participating in the two tests were not exactly the same. For more details about pigs’ rearing conditions see the Supplementary Material of the current study, the doctoral dissertation of Pérez Fraga^48^ and the ‘Online Resource 2’ belonging to the study of Gerencsér et al.^46^.

To compare pigs’ and dogs’ behaviour, 17 companion dogs were also tested once (9 intact and 2 neutered females, and 5 intact and 1 neutered males; mean ± SD age = 8.78 ± 0.77 months). Most of the dog owners were volunteers of the Family Dog Project (https://familydogproject.elte.hu/), and some of them applied for the tests through different social media platforms or questionnaires of the Family Dog Project. The socialisation background of the dogs was similar to that of the pigs. Our dog participants were of many different breeds (16 pure breeds: 1 Hungarian vizsla, 2 golden retriever, 1 Jack Russell terrier, 1 shih tzu, 2 American staffordshire terrier, 1 Tervueren, 2 beagle, 1 papillon, 1 mudi, 1 puli, 1 English cocker spaniel, 1 border collie, 1 labradoodle; and 1 mongrel). This increased the representativeness of our results to dogs in general. Importantly, prior to the experiment, pigs and dogs have never been tested in the SST and had no prior experience with the test location.

### Experimental setup

Here, we used a Strange Situation Test (SST) that consists of several episodes during which the subject is either alone, or with the potential attachment figure and/or a stranger in an unfamiliar environment. Based on Bowlby’s evolutionary approach^20^, this situation triggers specific patterns of attachment-related behaviours. In the SST, the stranger, together with the strange situation in the unfamiliar environment, and with the separation from the owner evoke moderate stress in the subjects^38^. The use of SST protocols involving the encounter with a stranger is common and well-established in dog SST experiments, yielding similar, robust, and replicable results across studies (for reviews see,^1,2,21^). The stress provoking effect of these protocols is confirmed by physiological studies^33,49–51^. Despite these, it is conceivable that in adult, well-socialised and friendly dogs/pigs the stranger may not remarkably increase the stress level even in the absence of the owner. But even in this case, basic differences in the subjects’ behaviours must be observable in the presence of the two persons, enabling the assessment of attachment.

**Figure 1:**
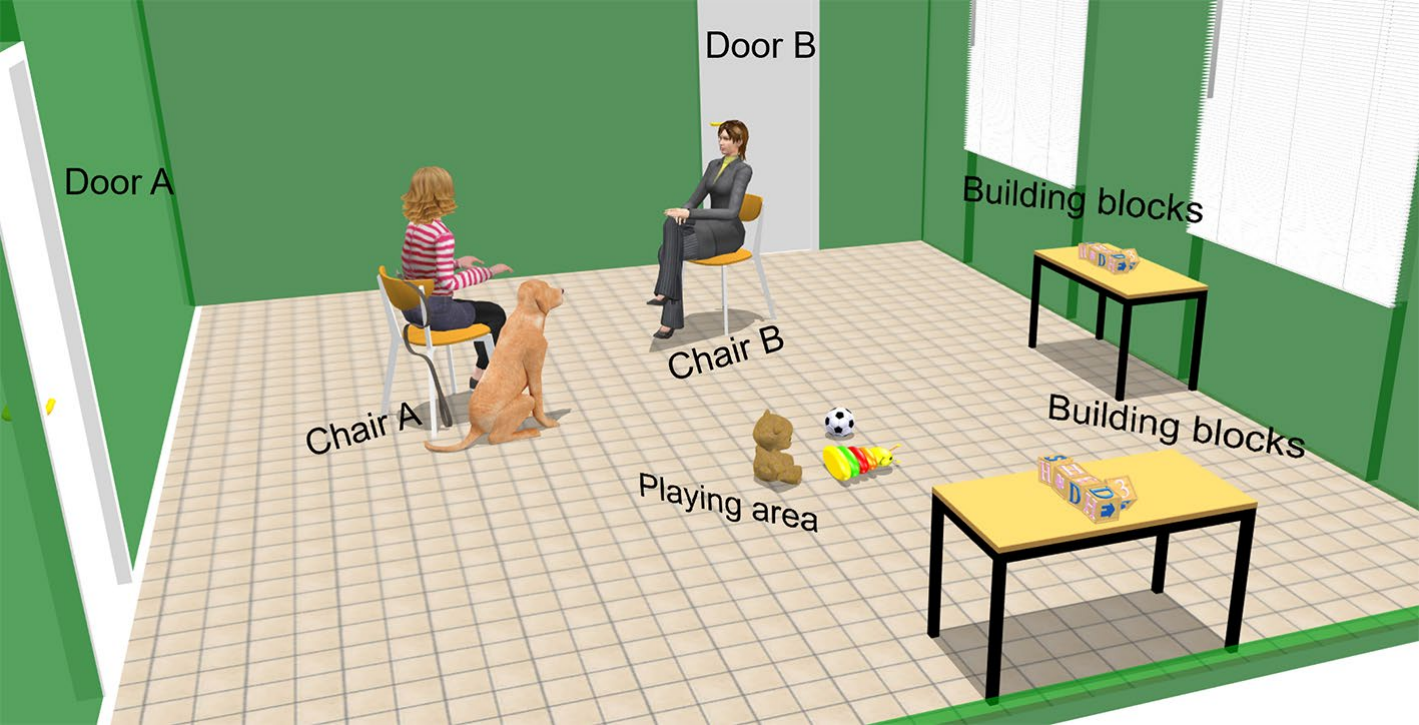
Experimental setting. Schematic drawing of the basic experimental setup of the Strange Situation Test.

Our SST protocol was based on the one used to investigate dog-owner attachment in the studies of Gábor et al.^3^ and Lenkei et al.^52^. The SST took place in the behavioural lab (6.27 m × 5.4 m) (Fig. 1) unfamiliar to the pigs and the dogs at the Department of Ethology, Eötvös Loránd University, Budapest, Hungary. Two chairs were placed in the middle of the room, facing each other, 1.5 m apart. The lab had two doors which were used by the stranger and the owner during the test (they always used the one closer to their assigned chair). The owner’s and the stranger’s chairs were randomised and balanced across tests. There were two additional chairs (for pigs) or tables (for dogs) with 6 wooden building blocks on them. These tables with the building blocks on them^3,52^ functioned as random objects for owners to perform actions neutral for their animals while ignoring them. This allowed us to observe if the owner's activity in themselves would affect the subjects’ proximity seeking behaviour, similarly to the case of human infants, who monitor the actions of the mother even if that is not directly related to them.

Various pet toys (e.g., balls) were placed in a playing area between the chairs. Subjects’ behaviour was recorded using four ceiling-mounted cameras strategically positioned to provide visibility of the entire lab. The same owner participated in both tests of the pigs, with the exception of one pig, who moved to another human family between the two SSTs and consequently changed its primary caregiver.

Importantly, excessively high levels of stress can result in extreme behaviours (i.e. panic or freezing) in which case attachment is not assessable. Pigs are prey animals whose stress level easily raises, so to increase their comparability with dogs (make their Anxiety scores similar), a “calming” box or blanket in which they usually sleep was placed at the corner of the room opposite the playing area. According to our previous experiences with pig testing and with the SST piloting, the lack of a calming object could have caused too high levels of stress in pigs as indicated by their extreme vocalisations or escaping behaviours. We assumed that the familiar scent of the calming box/blanket decreased the stress level of pigs.

### Protocol of the Strange Situation Test

The SST consisted of 6, 2-min-long episodes presenting the participants with differently stressful situations influenced by the presence and the absence of the owner and a stranger (Table 1). The owner and the stranger performed different activities the description of which can be found in Table 2. At the beginning of the test, owners got a blue-tooth headset playing a timed audio file with pre-recorded instructions, ensuring each activity to be performed in the given time by the owner. Strangers knew the protocol by heart and used a stopwatch to keep the timing. Before the test, an Experimenter welcomed the owners, provided them a detailed explanation of the process, and ensured that everything went well during the test. The experimenter was never the same person as the stranger, so both pigs and dogs met the stranger for the first time during the test.

**Table 1:** Detailed protocol of the Strange Situation Test.

Episode, duration (s)	Owner	Stranger
1 (4 × 30)	Sit	Absent
	Carry blocks	
	Sit	
	Pet/play with the pig/dog	
	Sit	Enter
2 (4 × 30)	Sit	Sit
		Carry blocks
		Sit
		Pet/play with the pig/dog
	Leave	Pet/play with the pig/dog
3 (4 × 30)	Absent	Sit
		Carry blocks
		Sit
		Pet/play with the pig/dog
	Enter + pet/play with the pig/dog	Leave
4 (4 × 30)	Sit	Absent
	Carry blocks	
	Sit	
	Pet/play with the pig/dog	
	Leave	
5 (60 + 2 × 30)	Absent	
		Enter the room then sit
		Sit
		Leave the room
6 (60 + 2 × 30)		Absent
	Enter the room then sit	
	Sit	
Experimenter enters the room–end of the test		

This table presents the detailed SST protocol based on the studies of Gábor et al.^3^ and Lenkei et al.^52^. For the detailed descriptions of the activities presented by the owner and the stranger see Table 2.

**Table 2:** Description of the activities performed by the owner (O) and the stranger (S) during the SST.

Activity	Description
Enter	O/S enters the room, stands on the door’s left side and waits a few seconds (~ 4) for the animals’ response. Then, in case the animal does not approach O/S on its own, they briefly and friendly greet it
Leave	O/S places a piece of their clothing on the chair’s back (e.g. scarf), then leaves the room through the door closer to the chair they sat on
Carry blocks	O/S is slowly walking between those two chairs which have the building blocks and the plastic top on them and carrying the blocks from one to the other. O/S is not allowed to interact with or look at the animal
Sit	O/S is sitting on their assigned chair (always on the same one within one test). O/S is allowed to look at the animal and pet it in case the animal initiates physical contact (jumps, leans, pushes nose)
Play/Pet	O/S initiates play or petting, depending on the willingness of the animal

This table shows the detailed description of the behaviours presented by the owner (O) and the stranger (S) during the SST of both pigs and dogs.

### Coding of behaviour

Based on the observed behaviour patterns, three major factors were formed and analysed; *Attachment* to the owner, *Anxiety* in the strange situation, and *Acceptance* of the stranger (willingness to interact with an unfamiliar person) (see^39^). This three-factor coding is often used in recent dog attachment papers (e.g.,^3,52^). For dogs, the same behaviours were coded as in the previous studies, but, for pigs, we made some slight modifications to better fit to their behaviour and emotional displays (Table 3). For example, normal tail wagging most times indicates positive emotions in both species^53,54^ while intense tail wagging or an increased tail wagging frequency is associated with stress in pigs^54,55^. In addition, in the SST context, dogs typically vocalise when stressed (except during playing and greeting). In contrast, pigs are a very vocal species^16^, thus their vocalisations were more carefully specified to ensure that we only code those that indicate stress^56–59^. Based on our observations from the past years with pigs, pigs’ reaction time is slower than that of dogs, which was also considered during the behaviour coding.

**Table 3:** Detailed description of the scoring of pigs’ and dogs’ behaviour in the SST.

Owner	Episode	Variable	Description of the scored behaviours	Score
Attachment				
Present	1, 2, 4, 6 (2nd min)	ProximityO	P/D is close to O (closest body part is within 1 m)—in most of the time when it does not explore or play	1
	1	ActiveO-1	During block-carrying, P/D watches or follows O for more than half of the time	1
	**2e**	**FollowO-1**	**When O leaves, P/D follows O to door (at least the front leg is within 1.5 m area) and orients towards the door**	**1**
	**4s**	**GreetO-1**	**When O enters P/D starts to approach immediately (P: as it notices the O coming in—without stopping; D: is within reach**	**1**
	4	ActiveO-2	During block-carrying, P/D watches or follows O for more than half of the time	1
	4e	FollowO-2	When O leaves, P/D follows O to door and orients towards the door—at least the front leg is within the 1.5 m area	0.5
	6s	GreetO-2	When O enters P/D starts to approach immediately (P: as it notices the O coming in—without stopping; D is within reach	0.5
Absent	**3**	**DoorS-1**	**P/D stands by or orients at O's door (for at least 5 s—score 0.5; almost all the time—score 1)**	**1**
	3	NoPlayS	P/D does not play with S although it played with her more than 10 s in Episode 2 (in O's presence)	1
	3, 5 (2nd min)	VocalS	P/D vocalises (D: except asking for ball from S or greet the O, P: loudly with high pitch AND/OR with high grunt frequency)	0.5
	3, 5	ChairS	P/D is at O’s chair for more than half of the time if it is not at the door	0.5
	5 (2nd min)	DoorS-2	P/D stands by or orients at O's door (for at least 5 s)	1
	5	EscapeS	When stranger enters, P/D first tries to sneak out through the door instead of greeting S	0.5
	6 (1st min)	Door	P/D stands by or orients at O's door (for at least 5 s)	0.5
Anxiety				
Present	**1**	**DoorO-1**	**P/D stands by or orients at any door (for at least 5 s—score 1, almost all the time during sit/play—score 2)**	**2**
	1, 2	SContactO	P/D seeks physical contact with O before the first separation from O	1
	1, 2, 4, 6 (2nd min)	VocalO	P/D vocalises (D: except asking for ball from S or greet the O, P: loudly with high pitch AND/OR with high grunt frequency)	1
	1, 2, 4	PassiveO	P/D does not play and does not explore for more than a few seconds (except for a pretty relaxed position, e.g., lies on the floor)	1
	1, 2, 4, 6 (2^nd^ min)	HideO	P/D stays (hides) under/behind O's chair (P: or at the box / carpet) for more than half of the time of the sit phases	1
	1, 2e, 4, 4e	LeadO	As soon as O stands up, P/D approaches door (going ahead of O) (4*0.5)	2
	4	DoorO-2	P/D stands by or orients at any door for at least 5 s	1
	**4s**	**FollowS**	**When S leaves, P/D follows S to door (at least the front leg is within the 1.5 m area) and orients towards the door**	**1**
	6 (2nd min)	DoorO-3	P/D stands by or orients at any door for at least 5 s	1
Absent	3	StressS	P stands motionless, including tails (~ freezing) OR vocalises loudly with high pitch AND/OR with increased grunt frequency OR pushes the door/box with its snout D runs around up and down for at least 10 s, or vocalises, or scratches door	1
	5, 6 (1st min)	Stress	P stands motionless, including tails (~ freezing) OR vocalises loudly with high pitch AND/OR with increased grunt frequency OR pushes the door/box with its snout D runs around up and down for at least 10 s, or vocalises, or scratches door (2 × 0.5)	1
	3	NoCalm-1	P/D does not play or lie down comfortably or explore for more than 10 s	1
	5, 6 (1st min)	NoCalm-2	P/D does not play or lie down comfortably or explore for more than 10 s	1
Acceptance				
Present	**1e**	**GreetS-1**	**When S enters P/D starts to approach immediately (P: as it notices S coming in—without stopping; D is within reach**	1
	1e	ContactS-1	P/D gets in physical contact with S and show no avoidance (retreat when touched), does not bite, D is wagging its tail	1
	2	ActiveS-1	During block-carrying, P/D watches or follows S for more than half of the time	1
	2	PlayS-1	P/D plays with S or is willing to be petted at least for 10 s (offers its body- > approaches S in reaching distance)	1
Absent	3, 5 (2nd min)	ProximityS	P/D stays close (closest body part is within reaching distance) to S in sit phases (at least for 5 s—1, almost all the time—2)	2
	3	ActiveS-2	During block-carrying, P/D watches or follows S for more than half of the time	1
	3	PlayS-2	P/D plays with S or is willing to be petted at least for 10 s (offers its body- > approaches S in reaching distance) (a little—1, a lot—2)	2
	5	Greet/ContactS-2	When S enters P/D starts to approach immediately (P: as it notices the S coming in—without stopping; D is within reach. P/D gets in physical contact with S and show no avoidance (retreat when touched), does not bite, D is wagging its tail	1
Any time	2, 3, 5	CloseS	P is mostly (for more than half of the time) close to the chair of S (within reaching distance) D offers the toy to stranger	1
	2, 3, 5	ContactS	P/D seeks physical contact (e.g., nudges or rubs to the legs of S) (any occurrence)	1
	2, 3	NoAvoidS	P/D does not avoid S during play (does not stand off, avoids her touch, bites, turns his back or face)	1

The table shows the description of the scored behaviours to assess Attachment, Anxiety and Acceptance. For description of SST episodes see Table 2. This scoring is adapted from the study of Gábor et al.^3^ and Lenkei et al.^52^. P: pig, D: dog, O: owner, S: stranger, s: start of the certain episode, e: end of the certain episode. Bold rows mark the 3 symmetrically coded variables between O and S.

### Analysis

One–one independent second coder coded > 10% of the pig and dog videos. From these, inter-rater reliability was calculated based on Cohen’s Kappa. This achieved 0.8–1 value indicating an excellent agreement in case of pigs, and 0.7–0.8 value indicating substantial agreement in case of dogs^60^.

To investigate whether pigs and dogs show specific patterns of attachment behaviours (i.e. they behave differently with the owner and the stranger in the SST context), the three symmetrically coded relevant variables between the owner and the stranger were compared similarly to the cat-dog comparative study of Gácsi et al. (submitted)^13^. These three variables were: (i) standing by the door in the presence of the owner (DoorO-1) vs. the stranger (DoorS-1) when the other person is absent, (ii) approaching the entering owner (GreetO-1) vs. stranger (GreetS-1) while the other person is in the room, and (iii) following the leaving owner (FollowO-1) vs. stranger (FollowS) while the other person stays in the room. In the case of both pigs and dogs, the number of individuals that showed/ did not show the relevant behaviour in the presence of the owner/stranger were compared via Related-Samples McNemar Change Test.

To compare the factor scores (Attachment, Anxiety, Acceptance) of the two species Mann–Whitney U tests or Independent-Samples t-Tests were used depending on the results of the Shapiro–Wilk normality test. To test the possible association between the factors in case of both pigs and dogs, Pearson correlation was used. All of the previous pig analyses were based on the data of the first pig SST to avoid the influence of a possible order effect. To test temporal stability of pigs’ behaviour across the first and the second SST, Pearson correlation and Wilcoxon signed-rank tests or Paired-Samples t-Tests were used depending on the results of the Shapiro–Wilk normality test. The pig who changed its owner between the two tests was also involved in these analyses. This decision was influenced by (1) the consideration that if this pig exhibited different attachment patterns, it would weaken our results, and (2) indirect evidence from the relevant dog literature, which indicates that dogs' attachment does not decrease following a change in ownership, as demonstrated by group-level results^26,51,61^.

IBS SPSS Statistics 23.0 was used for the statistical analyses.

### Ethics statement

The experiment was conducted at the behavioural lab of the Department of Ethology, Eötvös Loránd University, Budapest, Hungary. All applicable institutional, national and international guidelines for the use and care of animals were followed. The study was non-invasive and did not cause any harm to the animals. We state that the study is reported in accordance with ARRIVE guidelines. The local ethical committee (Állatkísérleti Tudományos Etikai Tanács, Pest Megyei Kormányhivatal Élelmiszerlánc-Biztonsági és Állategészségügyi Igazgatósága in Budapest, Hungary) approved the experiment (# PE/EA/430-6/2018). Pig and dog owners were volunteers, did not get any monetary compensation and gave written informed consent.

## Results

**Figure 2:**
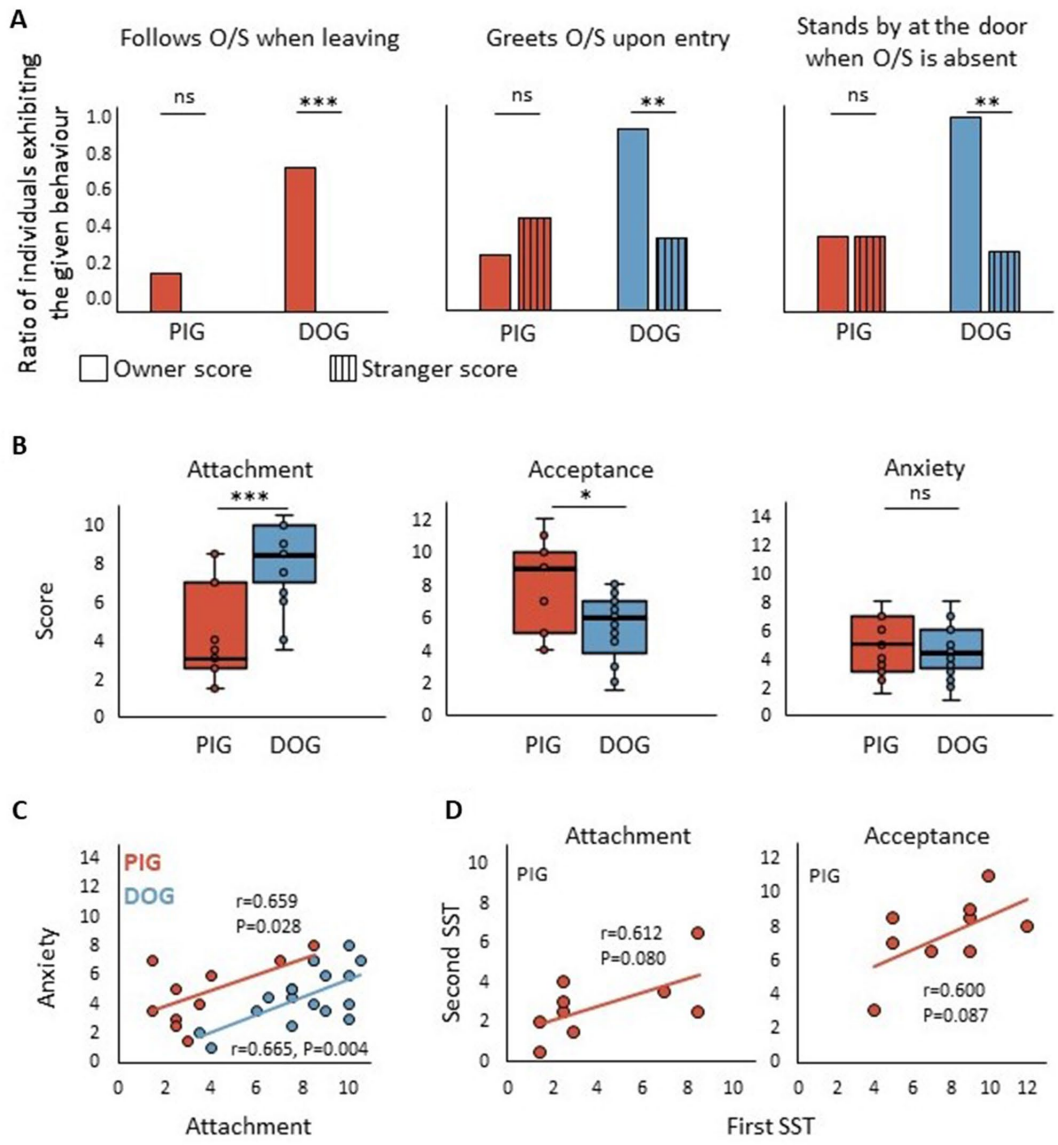
Results of the Strange Situation Tests. (**A**) Pigs’ and dogs’ behaviour along the three symmetrically coded variables between the owner and the stranger. For detailed description of the behaviours, see Table 3. (**B**) Pairwise comparison of pigs’ and dogs’ Attachment, Acceptance and Anxiety scores. (**C**) Attachment and Anxiety score correlation in pigs and dogs. (D) Similar tendency of pigs’ Attachment and Acceptance scores between their first and second SST. O: owner, S: stranger, SST: Strange Situation Test, ns: not significant, *P < 0.01, **P < 0.005, ***P ≤ 0.001. Dogs: N = 17 dogs (**A**–**D**); Pigs: N = 11 (**A**–**C**), D: N = 9 (**D**).

The comparison of the three symmetrically coded variables between the owner and the stranger revealed significant differences in dogs’ behaviour towards the two persons (Fig. 2A). More specifically, dogs were more likely to (1) stand by the door when the owner was absent and the stranger was present compared to the reverse situation (DoorS-1 vs. DoorO-1: df = 1, P = 0.003), (2) follow the leaving owner to the door while the stranger was present compared to the reverse situation (FollowO-1 vs. FollowS: df = 1, P < 0.000) and (3) approach the entering owner than the entering stranger while the other person was present (GreetO-1 vs. GreetS-1: df = 1, P = 0.004). None of these owner vs stranger comparisons revealed significant differences in pigs (DoorO-1 vs. DoorS-1: df = 1, P = 1.00; FollowO-1 vs. FollowS: df = 1, P = 0.50; GreetO-1 vs. GreetS-1: df = 1, P = 0.625).

The factor score comparison between the two species revealed significant differences in case of the Attachment (U = 163.0, P = 0.001) and Acceptance (t = 2.518, df = 26, P = 0.009) scores. Specifically, dogs had higher Attachment, while pigs had higher Acceptance scores than that of the other species. Such between species difference was not present in case of the Anxiety score (t = 0.585, df = 26, P = 0.282). For factor score details, see Fig. 2B and Table S1.

In both species, we found positive Attachment and Anxiety score correlations (Pig: r = 0.659, P = 0.028; Dog: r = 0.665, P = 0.004), but there was no association regarding the other two factors in either species (Attachment and Acceptance (Pig: r = − 0.332, P = 0.319, Dog: r = − 0.283, P = 0.271); Acceptance and Anxiety scores (Pig: r = 0.089, P = 0.795, Dog: r = 0.037, P = 0.888)) (Fig. 2C).

When testing the temporal stability of pigs’ behaviour in the SST, we found no differences between their first and second Attachment (Z = 7.500, P = 0.141), Anxiety (t = − 0.405, df = 8, P = 0.348) and Acceptance (t = − 0.236, df = 8, P = 0.410) scores indicating that their scores at the group level did not indicate a significant change between their first and second tests. Pearson correlation revealed that pigs’ Attachment (r = 0.612, P = 0.080) and Acceptance (r = 0.600, P = 0.087) scores, but not Anxiety scores (r = − 0.015, P = 0.970), showed a similar tendency in time (Fig. 2D).

## Discussion

In this study, by testing young individuals of two domesticated social species, we demonstrated differences between the nature of companion pig–owner and companion dog–owner relationships. Specifically, we found that in the Strange Situation Test (SST) (1) dogs but not pigs showed significantly different behaviours towards the owner and a stranger along three major attachment-specific variables, (2) dogs’ Attachment (towards the owner) score was higher than that of pigs, (3) pigs’ Acceptance (of the stranger) score was higher than that of dogs. Furthermore, (4) Attachment scores positively correlated with Anxiety scores in both species and (5) pigs’ Attachment and Acceptance scores showed a similar tendency in time. Our results demonstrate that, contrary to dogs, pigs typically do not show those behaviour patterns that characterize human infant-mother attachment in the SST.

This research contributes to the understanding of how interspecific attachment towards humans may evolve. The fact that dogs but not pigs showed a specific differentiation between the owner and the stranger indicate that only dogs’ relevant behaviours fulfilled the criteria of attachment. Considering that pigs and dogs are both domestic and social species, and that the tested individuals were kept similarly as companions, we can reject the hypothesis that domestication alone, or in interaction with the human family environment is enough to trigger companion animal–human attachment. The fact that pigs’ attachment scores did not increase between their first and second tests shows that even a longer period of time was not enough for the development of an attachment bond in pigs. Even though companion pigs and dogs occupy a very similar human social niche nowadays, only dogs have been under selective pressure to fit into this niche. Indeed, while for farm pigs the main selection criteria were excessive breeding and optimising meat stock^14^ and miniature pigs were later selected for medical research^15^, dogs were specifically bred for direct social interactions with humans^9^. These results coincide with the result of those cat studies revealing no owner-oriented attachment in cats^12,13^. That is, unlike dogs' artificial selection, the natural selection process^62^ and the different living conditions of domestic cats, might have not led to the degree of dependence on their owner necessary to develop a bond that fulfils all operational criteria of attachment^13^. In sum, our results suggest that dogs’ artificial selection that resulted in increased dependence on humans, facilitated the development of dogs’ human-analogue attachment towards their owner.

Differences between dogs’ and pigs’ owner-related behaviour in the SST may also stem from the distinct characteristics of their pre-domesticated ancestors. That is, the ways dogs and pigs form relationships with humans may partly be rooted in the intraspecific social world of their ancestors and can thus be linked to that of their closest living relatives, wolves and wild boars. Wolves possess a complex social network in which their fitness and survival are greatly reliant on cooperation with their mates for activities such as breeding and hunting. They reside in groups consisting of closely related family members of both sexes in which individuals depend on each other and are attached to the pack^63^. Regarding their relationship with humans, it is important to note that even intensely socialised wolves do not form attachment to their caregiver^8,64^. They, however, do exhibit signs of stress when separated from their handler and seek contact upon reunion when they are on a leash^65^. Wolves’ special social world and ability to express behaviours that are also important in the attachment system might serve as a baseline that, in interaction with domestication, allows for the development of human–dog attachment bonds.

Wild boars, similarly to wolves, are renowned for their gregarious nature and intricate social structures^66^. They even exhibit a division of labour among individuals, particularly in the context of cooperative breeding. But, in contrast to wolf packs, wild boars’ group organisation is the classic form of fission–fusion societies in which females form matriarchal units with their offspring and the boars only adhere to the group during the mating season^67^. In addition, pigs’ foraging strategy is centred around locating food patches^68^ during which individuals rely more on themselves. Due to these between-species differences, wild boars may not exhibit the same high degree of dependence on their mates as wolves. Thus, the ancestors of wild boars may have possessed less potential than the ancestors of wolves to, following their domestication, become socially attached partners of humans, which could also contribute to the different owner-directed behaviours of pigs and dogs in the SST context.

As early experiences with humans influence adult domestic animals’ (including dogs’ and pigs’) human-oriented behaviours^69,70^, one may argue that the divergence between pigs’ and dogs’ behaviour in the SST could be caused by the different lengths of their socialisation-sensitive periods. Dogs’ socialisation-sensitive period to humans seems to be somewhat longer (between 3 and 13 weeks of age)^71,72^ than that of pigs (between 2 and 10 weeks of age)^73^. Our subjects were adopted by their human family no earlier than 8–10 weeks of age, which may mark the end of the socialisation-sensitive period for pigs and the middle of this period for dogs. So, one alternative explanation for pigs not forming an attachment bond could be the insufficient amount of interaction with their owner during the socialisation-sensitive period. However, before adoption all subjects had extended experiences with humans, and early experience with the owner specifically is not required for the formation of attachment in dogs. Indeed, dogs acquired later than 3 months of age can also develop an attachment bond^2^. Even pet dogs living with a second caregiver^26^, adult shelter dogs^74^, and assistant dogs raised by a puppy walker prior to being placed with a disabled person^51,61^ can form attachment with their new owners. Therefore, differences in pigs’ and dogs’ human-oriented attachment are unlikely to be caused by different experiences with their owners in the sensitive period.

Even though our group-level results suggest that forming attachment to humans is not a general feature of companion pigs, we do not claim that its development is impossible on the individual level. In fact, there were a few pigs that got high attachment scores (i.e. similar to those of most dogs), at least in their first test. These pigs exhibited most of the behaviours specific to attachment measurable in the SST context. Individual variability in the attachment behaviour of both dogs^75^ and humans^27,76^ is most commonly attributed to differences in the individuals’ previous experiences with the attachment figure. While the environment of pig participants was controlled, and their owners handled them similarly, there is a possibility that some owners may have provided more security to the animals in ambiguous situations, potentially contributing to higher attachment scores. However, if this was the case, it should have resulted in a further increase in attachment scores for the second SST, which was not observed for either pig who got outstandingly high attachment scores in the first SST. Although some genetic variability could have played a role in the attachment behaviour of the pigs, the effect of differences in the selection forces that shaped the development of miniature pig variants involved in this study is unlikely. Each line of miniature pigs was selected for medical purposes^15^. This is further supported by indirect evidence showing that attachment behaviour of dogs is similar across breed types^52^. As, based on our sample size, we cannot draw firm conclusions on the causes of individual differences in pigs’ behaviour in the SST, thus future studies are required to reveal how developmental and inherited factors might form the bond between companion pigs and their owners. Our study raises the possibility that certain pigs may have the potential to show similar attachment behaviours towards their owners in the SST context as most dogs do.

The relative stability of individual pigs’ owner- and stranger-related behaviours, and that none of the group level factor scores increased or decreased across the two tests support the stability and repeatability of the SST in pigs. These findings also suggest that pigs’ willingness to interact with unfamiliar people and social relationship with the owner are relatively stable over time. While it seems that companion pigs typically do not show attachment to humans, some kind of social bond to their owners is indicated by the positive correlation between Anxiety and Attachment scores: higher levels of situational stress in pigs, just as in dogs, led to increased proximity-seeking behaviours. This may suggest that the owner can provide a certain level of security to the animals, as it was also observed in horses^77^. Proximity-seeking behaviour in stressful situations is one feature of but does not imply attachment; it has also been observed in other social bond types, especially in young individuals^78^. Pigs’ willingness to interact with unfamiliar humans as well is shown by their higher acceptance scores as compared to those of dogs. The relative temporal stability of pigs’ acceptance score suggests that pigs’ willingness to interact with unfamiliar humans was unlikely to be related to individual experiences; instead, it was probably due to some species-specific traits, such as neophilia^79^. That is, the high amount of time pigs spent in the vicinity of the stranger was likely part of their exploratory behaviour^42^.

Notwithstanding that companion pig-dog comparative experiments hold great potential for breakthroughs in understanding the origins and development of interspecific social relationships and communicative skills, their limitations must also be taken into account. (1) One may argue that the same behaviours may reflect different inner states in pigs and dogs which makes their direct comparison questionable. We, however, took special care to fit behaviours to emotional displays accurately and provided functionally matching behaviour descriptions for pigs and dogs (for details, see the “Methods” section). Importantly, as attachment is characterised by highly similar behavioural components across species (e.g.,^80^), this functional matching of behaviours was the most straightforward when scoring Attachment. (2) As the number of companion pigs is much lower than that of companion dogs, comparative studies present a trade-off between control over environmental similarity and sample size. In the current study, optimising for comparability, we opted for using companion pigs participating in our long-term research project, verifiably kept under conditions highly similar to that of dogs—and this resulted in a relatively low sample size. (3) Differentiating between passive anxious and passive relaxed behaviours is challenging in both pigs and dogs, potentially leading to slight inaccuracies in the Anxiety scores. To mitigate this, no Anxiety scores were given to animals in passive but comfortable positions (e.g., lying on the floor). (4) To equalise the Anxiety scores of pigs and dogs to an appropriate, moderate level, we introduced a calming box/blanket for pigs, but not for dogs, the presence or scent of which might have impacted their Attachment scores and Acceptance-related behaviours differently, even without direct interaction with this object. However, the facts that pigs’ and dogs’ Anxiety scores did not differ significantly, that all five pigs who received scores in the HideO measure consistently stayed (hid) under/behind O's chair and none of them did so at the calming box/blanket for more than half of the time of the sit phases support that this design difference had no significant direct effect on between-species differences of Attachment or Acceptance scores.

In sum, our results show that despite the many similarities of companion pigs and dogs—such as living conditions, similar role in human families, intense exposure to human social stimuli, and belonging to domesticated, vocal, social and group-living species—the nature of companion pig-owner and dog-owner relationships differ significantly. The special human analogue attachment relationship of dogs with their owners does not seem to appear in companion pigs as a general feature. We conclude that domestication and intense exposure to human social stimuli during development are not sufficient to trigger attachment towards humans in companion animals. This suggests that, along with certain species predispositions, the specific selection that had led to increased dependence on humans was an essential component for the evolutionary emergence of companion animal-human attachment.

### Supplementary Information

Supplementary Information.

## Data Availability

The raw data of the study are available at the following link: https://docs.google.com/spreadsheets/d/1ogQBzQi8Q0tGQ1Kf5B8CeHYLNL1MCYRU/edit#gid=1520133776.

## References

[1] Prato Previde E, Valsecchi P (2014). The immaterial cord: The dog-human attachment bond. Soc. Dog Behav. Cogn..

[2] Topál, J. & Gácsi, M. Lessons we should learn from our relationship with dogs: an ethological approach. In *Crossing Boundaries Investig. Human-Animal Relationships* (2012).

[3] Gábor A (2021). Social relationship-dependent neural response to speech in dogs. Neuroimage.

[4] Mariti C, Ricci E, Zilocchi M, Gazzano A (2013). Owners as a secure base for their dogs. Behaviour.

[5] Topál J (2005). Attachment to humans: A comparative study on hand-reared wolves and differently socialized dog puppies. Anim. Behav..

[6] Hansen Wheat C, Larsson L, Berner P, Temrin H (2022). Human-directed attachment behavior in wolves suggests standing ancestral variation for human–dog attachment bonds. Ecol. Evol..

[7] Hall NJ, Lord K, Arnold AMK, Wynne CDL, Udell MAR (2015). Assessment of attachment behaviour to human caregivers in wolf pups (*Canis lupus lupus*). Behav. Processes.

[8] Gácsi M, Miklósi Á, Topál J (2023). Comment on “Human-directed attachment behaviour in wolves suggests standing ancestral variation for human-dog attachment bonds” Dogs are unique. Ecol. Evol. Press..

[9] Miklosi, A. *Dog Behaviour, Evolution, and Cognition*. *Dog Behaviour, Evolution, and Cognition* (2015).

[10] Vitale KR, Behnke AC, Udell MAR (2019). Attachment bonds between domestic cats and humans. Curr. Biol..

[11] Edwards C, Heiblum M, Tejeda A, Galindo F (2007). Experimental evaluation of attachment behaviors in owned cats. J. Vet. Behav. Clin. Appl. Res..

[12] Potter A, Mills DS (2015). Domestic cats (*Felis silvestris catus*) do not show signs of secure attachment to their owners. PLoS ONE.

[13] Gácsi, M., Uccheddu, S., Csepregi, M. & Miklósi, Á. Only dogs show attachment behaviour toward their owners: A comparative study on cats and dogs. (**submitted**).

[14] Frantz L (2016). The evolution of suidae. Annu. Rev. Anim. Biosci..

[15] Vodička P (2005). The miniature pig as an animal model in biomedical research. Ann. N. Y. Acad. Sci..

[16] Marino, L. & Colvin, C. M. Thinking pigs: A comparative review of cognition, emotion, and personality in *Sus domesticus*. *Int. J. Comp. Psychol.* **28** (2015).

[17] Sroufe LA, Waters E (1977). Attachment as an organizational construct. Child Dev..

[18] Wickler W (1976). The ethological analysis of attachment: Sociometric, motivational and sociophysiological aspects. Z. Tierpsychol..

[19] Simpson JA, Rholes WS (2000). Caregiving, attachment theory, and the connection theoretical orientation. Psychol. Inq..

[20] Bowlby, J. *Attachment and loss*. *Attachment* vol. 1 (1969).

[21] Nagasawa M, Mogi K, Kikusui T (2009). Attachment between humans and dogs. Jpn. Psychol. Res..

[22] Payne E, Bennett PC, McGreevy PD (2015). Current perspectives on attachment and bonding in the dog–human dyad. Psychol. Res. Behav. Manag..

[23] Strathearn L (2011). Maternal neglect: Oxytocin, dopamine and the neurobiology of attachment. J. Neuroendocrinol..

[24] Waters E, Beauchaine TP (2003). Are there really patterns of attachment? Theoretical and empirical perspectives. Dev. Psychol..

[25] Doyle C, Cicchetti D (2017). From the cradle to the grave: The effect of adverse caregiving environments on attachment and relationships throughout the lifespan. Clin. Psychol. Sci. Pract..

[26] Gácsi M (2003). A kutyák gazda iránt mutatott kötődési viselkedésének etológiai vizsgálata.

[27] Rajecki DW, Lamb ME, Obmascher P (1978). Toward a general theory of infantile attachment: A comparative review of aspects of the social bond. Behav. Brain Sci..

[28] Lorenz, K. *King Solomon’s Ring*. *King Solomon’s Ring*.10.4324/9780203165966 (2003).

[29] Lehman EB, Denham SA, Moser MH, Reeves SL (1992). Soft object and pacifier attachments in young children: The role of security of attachment to the mother. J. Child Psychol. Psychiatry.

[30] Konok V, Pogány Á, Miklósi Á (2017). Mobile attachment: Separation from the mobile phone induces physiological and behavioural stress and attentional bias to separation-related stimuli. Comput. Human Behav..

[31] Harlow HF (1958). The nature of love. Am. Psychol..

[32] Gácsi M, Maros K, Sernkvist S, Faragó T, Miklósi Á (2013). Human analogue safe haven effect of the owner: Behavioural and heart rate response to stressful social stimuli in dogs. PLoS ONE.

[33] Palestrini C, Previde EP, Spiezio C, Verga M (2005). Heart rate and behavioural responses of dogs in the Ainsworth’s Strange Situation: A pilot study. Appl. Anim. Behav. Sci..

[34] Ryan MG, Storey AE, Anderson RE, Walsh CJ (2019). Physiological indicators of attachment in domestic dogs (*Canis familiaris*) and their owners in the strange situation test. Front. Behav. Neurosci..

[35] Clay AW, Bloomsmith MA, Bard KA, Maple TL, Marr MJ (2015). Long-term effects of infant attachment organization on adult behavior and health in nursery-reared, captive chimpanzees (*Pan troglodytes*). J. Comp. Psychol..

[36] Simonelli A (2014). Interactive behaviors and attachment patterns in the strange situation procedure: A validation of the Ainsworth model. Psychol. Behav. Sci..

[37] Van Rosmalen L, Van der Veer R, Van der Horst F (2015). Ainsworth’s strange situation procedure: The origin of an instrument. J. Hist. Behav. Sci..

[38] Ainsworth MDS, Wittig BA (1969). Attachment and the exploratory behaviour of one-year-olds in a strange situation. Determ. Infant Behav..

[39] Topál J, Miklósi Á, Csányi V, Dóka A (1998). Attachment behavior in dogs (*Canis familiaris*): A new application of Ainsworth’s (1969) strange situation test. J. Comp. Psychol..

[40] Carreiro C, Reicher V, Kis A, Gácsi M (2022). Attachment towards the owner is associated with spontaneous sleep EEG parameters in family dogs. Animals.

[41] Kovács K (2018). Dog-owner attachment is associated with oxytocin receptor gene polymorphisms in both parties. A comparative study on Austrian and Hungarian border collies. Front. Psychol..

[42] Terlouw EMC, Porcher J (2005). Repeated handling of pigs during rearing. I. Refusal of contact by the handler and reactivity to familiar and unfamiliar humans. J. Anim. Sci..

[43] Hemsworth PH, Barnett JL (1992). The effects of early contact with humans on the subsequent level of fear of humans in pigs. Appl. Anim. Behav. Sci..

[44] Brajon S (2015). Persistency of the piglet’s reactivity to the handler following a previous positive or negative experience. Appl. Anim. Behav. Sci..

[45] Bensoussan S, Tigeot R, Lemasson A, Meunier-Salaün M-C, Tallet C (2019). Domestic piglets (*Sus scrofa domestica*) are attentive to human voice and able to discriminate some prosodic features. Appl. Anim. Behav. Sci..

[46] Gerencsér L, Pérez Fraga P, Lovas M, Újváry D, Andics A (2019). Comparing interspecific socio-communicative skills of socialized juvenile dogs and miniature pigs. Anim. Cogn..

[47] Pérez Fraga P, Gerencsér L, Lovas M, Újváry D, Andics A (2021). Who turns to the human? Companion pigs’ and dogs’ behaviour in the unsolvable task paradigm. Anim. Cogn..

[48] Pérez Fraga P (2023). Interspecific Socio-communicative Abilities of the Family Dog and the Family Pig from a Comparative Ethological Perspective.

[49] Fallani G, Prato Previde E, Valsecchi P (2007). Behavioral and physiological responses of guide dogs to a situation of emotional distress. Physiol. Behav..

[50] Mongillo P (2013). Does the attachment system towards owners change in aged dogs?. Physiol. Behav..

[51] Valsecchi P, Previde EP, Accorsi PA, Fallani G (2010). Development of the attachment bond in guide dogs. Appl. Anim. Behav. Sci..

[52] Lenkei R, Carreiro C, Gácsi M, Pongrácz P (2021). The relationship between functional breed selection and attachment pattern in family dogs (*Canis familiaris*). Appl. Anim. Behav. Sci..

[53] Tami G, Gallagher A (2009). Description of the behaviour of domestic dog (*Canis familiaris*) by experienced and inexperienced people. Appl. Anim. Behav. Sci..

[54] Marcet Rius M (2018). Tail and ear movements as possible indicators of emotions in pigs. Appl. Anim. Behav. Sci..

[55] Camerlink I, Ursinus WW (2020). Tail postures and tail motion in pigs: A review. Appl. Anim. Behav. Sci..

[56] Reimert I, Bolhuis JE, Kemp B, Rodenburg TB (2013). Indicators of positive and negative emotions and emotional contagion in pigs. Physiol. Behav..

[57] Briefer EF (2022). Classification of pig calls produced from birth to slaughter according to their emotional valence and context of production. Sci. Rep..

[58] Friel M, Kunc HP, Griffin K, Asher L, Collins LM (2019). Positive and negative contexts predict duration of pig vocalisations. Sci. Rep..

[59] Linhart P, Ratcliffe VF, Reby D, Špinka M (2015). Expression of emotional arousal in two different piglet call types. PLoS ONE.

[60] Cohen J (1960). A coefficient of agreement for nominal scales. Educ. Psychol. Meas..

[61] Fallani G, Previde EP, Valsecchi P (2006). Do disrupted early attachments affect the relationship between guide dogs and blind owners?. Appl. Anim. Behav. Sci..

[62] Driscoll CA, Macdonald DW, O’Brien SJ (2009). From wild animals to domestic pets, an evolutionary view of domestication. Proc. Natl. Acad. Sci. USA.

[63] Coulter MW, Mech LD (1971). The wolf: The ecology and behavior of an endangered species. J. Wildl. Manag..

[64] Gácsi M (2005). Species-specific differences and similarities in the behavior of hand-raised dog and wolf pups in social situations with humans. Dev. Psychobiol..

[65] Lenkei R, Újváry D, Bakos V, Faragó T (2020). Adult, intensively socialized wolves show features of attachment behaviour to their handler. Sci. Rep..

[66] Maselli V (2014). Wild boars’ social structure in the Mediterranean habitat. Ital. J. Zool..

[67] Signoret, J. P., Baldwin, B. A., Fraser, D. & Hafez, E. S. E. The behaviour of swine. In *Behaviour of Domestic Animals* 295–329 (1975).

[68] Thomsen LR, Nielsen BL, Larsen ON (2010). Implications of food patch distribution on social foraging in domestic pigs (*Sus scrofa*). Appl. Anim. Behav. Sci..

[69] Lenkei R, Pogány Á, Fugazza C (2019). Social behavior in dog puppies: Breed differences and the effect of rearing conditions. Biol. Futur..

[70] Hemsworth PH, Barnett JL, Hansen C, Gonyou HW (1986). The influence of early contact with humans on subsequent behavioural response of pigs to humans. Appl. Anim. Behav. Sci..

[71] Freedman DG, King JA, Elliot O (1961). Critical period in the social development of dogs. Science.

[72] Scott JP, Fuller JL (2019). Genetics and the social behaviour of the dog. Genet. Soc. Behav. Dog.

[73] D’Eath RB (2005). Socialising piglets before weaning improves social hierarchy formation when pigs are mixed post-weaning. Appl. Anim. Behav. Sci..

[74] Gácsi M, Topál J, Miklósi Á, Dóka A, Csányi V (2001). Attachment behavior of adult dogs (*Canis familiaris*) living at rescue centers: Forming new bonds. J. Comp. Psychol..

[75] Marinelli L, Adamelli S, Normando S, Bono G (2007). Quality of life of the pet dog: Influence of owner and dog’s characteristics. Appl. Anim. Behav. Sci..

[76] Crowell J, Fraley RC, Shaver PR, Cassidy J, Shaver PR (2008). Measures of individual differences in adolescent and adult attachment. Handbook of Attachment: Theory, Research, and Clinical Applications.

[77] Lundberg P, Hartmann E, Roth LSV (2020). Does training style affect the human-horse relationship? Asking the horse in a separation–reunion experiment with the owner and a stranger. Appl. Anim. Behav. Sci..

[78] Rault JL, Waiblinger S, Boivin X, Hemsworth P (2020). The power of a positive human–animal relationship for animal welfare. Front. Vet. Sci..

[79] Trickett SL, Guy JH, Edwards SA (2009). The role of novelty in environmental enrichment for the weaned pig. Appl. Anim. Behav. Sci..

[80] Gaskins S (2017). Meaning and methods in the study and assessment of attachment. Cult. Nat. Attachment.

