# Supplementary material for: Domestication and exposure to human social stimuli are not sufficient to trigger attachment to humans: a companion pig-dog comparative study
Source: Sci Rep. 2024 Jul 8;14:14058. doi: 10.1038/s41598-024-63529-3 (PMC11231355; doi:10.1038/s41598-024-63529-3)
Supplement: Supplementary file 1 — Supplementary Information. [file 41598_2024_63529_MOESM1_ESM.pdf]

Supplementary Material

Living conditions of pig participants and selection procedure of pig owners

Living conditions of pig participants were comparable to that of dogs. To ensure this, at the beginning of our long-term Family Pig Project, we implemented a strict selection procedure for owners who volunteered to participate and engaged in close cooperation with the Department of Ethology for several years. Pig owners were chosen from over a hundred applicants after completing a questionnaire and undergoing an interview. During the interview, we assessed the prospective living environment of the pigs to ensure that it met all welfare requirements. Additionally, we explained the requirements and obligations to prospective pig owners for enrolling in the project. These included the commitment to bring the pigs to the university for socialization, training, and participation in behavioural tests. A minimum of one visit per week was necessary for project participation. All pig owners maintained direct communication with researchers, having the means to contact them via email or phone for any concerns regarding pig behaviour or health. In addition, a dedicated Facebook group was created to facilitate picture sharing and keep all owners updated on the progress of the Family Pig Project. Importantly, we ensured that our pig owners had extended experience with dog keeping. Most of our pigs lived together with dogs within their human family and had similar access to the garden of the house they lived in as dogs do. All pigs regularly slept inside the house/flat, were regularly walked on leash, got used to variable environments and public places and to physical and social interactions with humans, were familiar with several rooms of the Department of Ethology, Eötvös Loránd University (Budapest, Hungary), and were experienced with public transport or being transported by car. (Gerencsér et al. 2019, Pérez Fraga 2023)

Table S1. Score details of pigs and dogs

	Attachment			Acceptance			Anxiety		
	DOG	PIG		DOG	PIG		DOG	PIG	
		1st	2nd		1st	2nd		1st	2nd
mean	7.97	4.09	2.80	5.41	7.73	7.30	4.50	4.95	5.05
SEM	0.50	0.80	0.52	0.49	0.86	0.93	0.46	0.66	0.92
SD	2.07	2.64	1.64	2.01	2.87	2.95	1.89	2.18	2.92

The Table shows the mean, SEM and SD of pigs' and dogs' Attachment, Acceptance and Anxiety scores.

References

Gerencsér, L., Pérez Fraga, P., Lovas, M., Újváry, D. & Andics, A. (2019). Comparing interspecific socio-communicative skills of socialized juvenile dogs and miniature pigs. Anim. Cogn. https://doi.org/10.1007/s10071-019-01284-z.

Pérez Fraga P. (2023). Interspecific socio-communicative abilities of the family dog and the family pig from a comparative ethological perspective. Doctoral dissertation (Eötvös Loránd University, Budapest, Hungary). https://doi.org/10.15476/ELTE.2023.101.
